# The General Hospitals of the Territorial Army

**Published:** 1912-09-21

**Authors:** 


### THE ROYAL ARMY MEDICAL CORPS SECTION.

### The General Hospitals of the Territorial Army.

In our issues of The Hospital for June 13 and July 11, 1908, we described the General Hospitals of the Territorial Army, and indicated that the provision of buildings and equipment for them, as well as a certain proportion of their staff, had, at the request of the War Office, been handed over by the different County Associations concerned to the British Red Cross Society, wherever its local branches would undertake the duty. These hospitals, it will be remembered, are 23 in number, and are located in our large towns and cities, but preferably in those where schools of medicine exist. They are to contain 520 beds, of which 20 are reserved for officers, and are intended for the reception and medical care of the sick and wounded from the front, and they are to be of such a nature and so equipped that there will be obtained in them the same skilful treatment and comforts that are afforded by civil hospitals of the first rank. All the arrangements for the formation of these hospitals were given in a special Circular Memorandum issued in May 1908, and also in the original schemes for the organisation of Voluntary Aid in England and Wales and in Scotland.

### The Red Cross Society's Position.

The majority of the Red Cross Committees fell in with the War Office proposal, and agreed to undertake the work, and since 1908 they have had the matter in hand, some of them having even selected the necessary buildings, and arranged for their equipment as well as for the services of the extra 66 privates required for carrying on the general work of the hospital when opened. In certain cases, too, the approval of the War Office for the arrangements made have been obtained.

When the military authorities first made their proposal to the Red Cross Council we expressed the opinion that its acceptance might be beneficial to the Red Cross Society, as it would bring it into notice and into touch generally with the people; while it would also be advantageous to the hospitals themselves, as under the scheme they would become more of a living reality than they probably would be under the Territorial régime, where financial difficulties would prevent a great deal being done for them. At the same time we recognised that the idea had its drawbacks, for it gave the hospitals the appearance of being Red Cross units when in reality they were Territorial ones, and as such entitled to be provided for out of Territorial funds. The Red Cross Committees, however, soon found that no monetary grant was forthcoming and that they had to provide the means for meeting any financial expenditure incurred, for even in peace time initial organisation means outlay, unless the matter in hand is to be only a paper scheme. As an outcome of this there arose the feeling that Red Cross funds should not be employed in doing Territorial work, consequently a good deal of apathy prevailed in connection with the provision of these General Hospitals, the Red Cross Committees moving rather half-heartedly in dealing with the matter.

In one or two instances an effort was made to utilise the Poor-Law hospitals in some of our big cities on the basis of arranging with the authorities that a certain number of beds would be handed over within a certain time and at a cost per bed on the occurrence of the mobilisation of the Territorial Army, thus obviating any preliminary expenditure. The proposal was entertained, we understand, by the parochial authorities in the case of the Edinburgh and Glasgow General Hospitals, and, if it is successfully carried through, it seems to us that, if the accommodation can be spared and terms agreed upon, it is a reliable, efficient, and economical way of solving a very important and somewhat difficult problem.

### The New Regulations.

Quite recently, however, the War Office has issued an entirely new set of regulations on the subject of the Territorial General Hospitals, and in these they are treated as military units, and the County Territorial Associations are called upon to make during peace time the entire arrangements for their formation. In short, by these new instructions the onus of providing these hospitals is removed from the Red Cross Committees and is placed on the Territorial Associations, whose duty it undoubtedly is, just as much as is the raising of the field units of the Territorial Medical Service. If the County Associations, however, are wise, they will avail themselves of any preparations already made by the Red Cross Committees, because in some cases a great deal of useful work has been done by the latter during the four years that the formation of these hospitals was left to them.

A perusal of the new regulations shows that no change has been made in the stations where the General Hospitals are to be established in the event of the mobilisation of the Territorial Force, and such hospitals will contain as before 520 beds, 20 being reserved for officers.

### The Hospital Staff.

The permanent *personnel* of each hospital is still to consist of three officers (who will act respectively as administrator, registrar, and quartermaster) and of forty-three other ranks, who will undergo their annual training, either in a selected Military Hospital or in a R.A.M.C. Territorial Force School of Instruction, or in any other selected institution. No change has been made in the numbers, conditions of service, and retirement of the remaining officers of each hospital. They will comprise four lieutenant-colonels, eight majors, and twenty captains with substantive rank. They will undergo no training in peace, and they will be selected, as formerly laid down, from the leading members of the medical profession. They will be appointed as officers whose services will be available on mobilisation for performing the clinical and other duties associated with the General Hospitals when they are opened and are receiving patients. The necessary nursing staff will be supplied by the authorised establishment of the Territorial Force Nursing Service. To complete the subordinate *personnel* of each hospital sixty-six privates are needed, twenty for ward duties, twenty-nine as bâtmen, eleven for general duties, and six as supernumeraries. The bâtmen and general duty men will be employed as porters, liftmen, and messengers, and also for work in the laundry.

### The Effect of Mobilisation.

On the mobilisation of the hospital these 66 men will be enlisted into the Royal Army Medical Corps Territorial Force, when they will be clothed and equipped as in the case of other soldiers of the Territorial Force, their regimental pay and allowances being the same as that of the members of the R.A.M.C. (T.F.). The War Office suggests that Territorial County Associations should approach the Red Cross County Directors who have charge of the Voluntary Aid Detachments with the view of their registering the names of male members willing to enlist on mobilisation of the Territorial Force for service in the Territorial General Hospitals, and in this way obtain the sixty-six men wanted. We must say that this proposed procedure does not recommend itself to us, as it violates a well-recognised principle that no man should serve in a dual capacity in any scheme of Home Defence, and we think that the suggested arrangement, if adopted, will only lead to confusion. It is to the Veteran Reserve that the County Associations should look for the supply of these men, and they can do so, we believe, with every prospect of obtaining them. In the event of the hospital being called up for service, it will be mobilised just as are the other medical units, by the Assistant Director of the Medical Services (known formerly as the Administrative Medical Officer) of the division in whose area the General Hospital is.

The regulations are very clear and emphatic that the reponsibility for the preparation in peace time of the scheme for the formation of each General Hospital rests with the General Officer Commanding-in-Chief, and that he must associate himself with the County Association responsible for the hospital or hospitals in the area under consideration. Further, that the scheme should be drawn up under the guidance of the Assistant Director of Medical Services for the division in whose area the General Hospital is situated, and that there should be associated with him his Divisional Sanitary Officer, the Administrator, and the principal Matron of the Hospital.

### Selection and Equipment.

The scheme should deal with the selection of suitable buildings for the hospitals and with the provision of the equipment, this latter being supplied by the County Association concerned, who should rely, it is suggested, as far as possible upon local resources for its supply and should take as a guide the Medical and Surgical Equipment of a General Hospital of the Expeditionary Force, the details of which are furnished in a special appendix. Each scheme should include rough plans of the proposed buildings, with the necessary re-appropriations shown, and when completed must be submitted for the approval of the Army Council, it being also laid down that even after approval it must be revised annually. After a careful perusal of these recently issued regulations we are of opinion that, if really carried out, they will place the General Hospitals on a much more satisfactory footing and will ensure their being more rapidly mobilised, as well as being found more efficient when they are so.

